# Participants’ experiences of AVATAR therapy for distressing voices: a thematic qualitative evaluation

**DOI:** 10.1186/s12888-022-04010-1

**Published:** 2022-05-24

**Authors:** Mar Rus-Calafell, Nils Ehrbar, Thomas Ward, Clementine Edwards, Mark Huckvale, Jennifer Walke, Philippa Garety, Tom Craig

**Affiliations:** 1 Mental Health Research and Treatment Centre, Faculty of Psychology, Ruhr-Universität Bochum, Bochum, Germany; 2 Department of Health Service and Population Research, Institute of Psychiatry, King’s College London, Psychology & Neuroscience, London, UK; 3 Department of Psychology, Institute of Psychiatry, Psychology & Neuroscience, King’s College London, London, UK; 4 South London & Maudsley NHS Foundation Trust, London, UK; 5 Speech, Hearing and Phonetic Sciences, Division of Psychology and Language Sciences, University College London, London, UK

**Keywords:** AVATAR therapy, Auditory hallucinations, Psychosis, Qualitative study

## Abstract

**Background:**

AVATAR therapy is an innovative therapy designed to support people with distressing voices. Voice hearers co-create a digital representation of their voice and engage in dialogue with it. Although it has been successfully tested in a powered randomised controlled trial (ISRCTN65314790), the participants’ experience of this therapy has not been yet evaluated. We aimed to explore enablers and barriers to engagement with the therapy and potential for real-world impact on distressing voices.

**Methods:**

Thirty per cent of those who completed AVATAR therapy (15 people in total) and 5 who dropped out from therapy within the main AVATAR RCT were invited to participate in a semi-structured interview, which was audio-recorded and subsequently transcribed.

**Results:**

Fourteen therapy completers (28% of the full sample) and one person who dropped out of therapy after 1 active session, were interviewed. Thematic analysis was used to explore the interviews. A total of 1276 references were coded, and five overarching themes identified: AVATAR therapy set-up; voice embodiment and associated emotions; working in a safe space (supported by the therapist); learning new ways of relating to the voices; impact of therapy on everyday life. Overall, the therapy set-up, with its digital components and its distinctive features as compared with common face-to-face talking therapies, was satisfactory. The inclusion of technology was well accepted as both a means to deliver the therapy and a tool to create a digital representation of the person’s distressing voice. The co-creation of the avatar and the enactment of the relationship between the person and the voice were perceived as a very helpful process to promote the therapeutical dialogue. Participants reported engaging well with the therapist and feeling supported and identified specific learnt strategies to deal with the voices and how they have had an impact on everyday life.

**Conclusions:**

AVATAR therapy is acceptable and provides benefit for participants with psychosis. Our results highlighted the enablers and challenges of working dialogically with distressing voices using a digital representation and dealing with highly demanding emotional, cognitive, and relational processes linked to the experience. Our analysis also identified the core strategies learnt by participants and how these were generalised to their daily life resulting into a positive change in different domains, and in particular broader social relationships.

## Background

Emerging psychological therapies for distressing voices in psychosis target the relationship between the person and their voice in order to reduce the impact and distress that negative voices can have on people [1–3]. AVATAR therapy is an innovative therapy approach designed to support voices hearers to develop an increased sense of power and control over their voices by promoting a dialogue between the person and a digital representation of the voice (or avatar) [4]. Two therapy phases are involved: Phase 1 (sessions 1 to 3) focuses on exposure and assertive responding; Phase 2 (sessions 4 to last) focuses on relational, emotional, and developmental processes [4, 5]. Preliminary encouraging evidence for the efficacy of the therapy has been reported in two independent pilot studies [6, 7] and further supported by the findings of a large fully-powered randomised controlled trial comparing AVATAR therapy and Supportive Counselling [8], which showed a post-therapy effect size of d = 0.8 on the primary outcome (total score on the Psychotic Symptom Rating Scales, auditory hallucinations subscale (PSYRATS–AH, [9]). AVATAR therapy is now being further examined for optimal delivery, effectiveness and cost-effectiveness in a new multi-centre randomised controlled trial ISRCTN55682735 [10].

While AVATAR therapy shares a common theoretical rationale with other cognitive and relational therapies for voices it also involves a number of distinctive elements. Firstly, it requires the person to create a digital visual representation of their voice, matching physical and voice characteristics, using bespoke computer software. This unique process of virtual voice embodiment and real-time communication involves direct exposure to the anxiety-provoking fear stimuli and the experience of sense of voice presence (i.e., the degree to which the avatar dialogue matched the everyday voice experience [11]) during sessions. Secondly, the therapy set-up requires that the person and the therapist move to separate rooms during therapy dialogues (as the therapist is in control of voicing the avatar). Thirdly, all sessions are audio recorded and a copy of the avatar dialogue is provided on an MP3 player to the participant with instructions to listen to the recording at home, especially when they hear the voice(s).

These distinctive aspects are linked to the blending of digital technology within this face-to-face relational therapy. Some studies on digital interventions have highlighted the importance of exploring the effects of digital technology on therapeutic alliance [12]. One of the key aspects is the role of the human professional support when delivering digital interventions [13]. In AVATAR therapy, two essential delivery processes are directly linked to the role of the therapist: 1) voicing the avatar (which includes use of derogatory verbatim content in early sessions) and 2) providing continuous support and encouragement to the voice hearer to stand up and talk back to the avatar using assertive responding [5].

Different aspects of AVATAR therapy have been examined, including its efficacy [8], key therapy targets [5], reduction of subjective in-session anxiety levels and role of sense of voice presence [11],and changes in dialogue dynamics and content [14]. However, a qualitative exploration of the person’s experience of AVATAR therapy is crucial to better understand the intervention’s perceived helpfulness and impact, and to optimise its delivery. The present study is the first to explore the personal experience of taking part in AVATAR therapy within a full powered randomised trial, to further understand the impact and experience of this therapy directly from people having this therapy.

## Methods

The present study was part of the AVATAR trial (ISRCTN65314790), a randomised clinical trial based in London (UK) which commenced in November 2013 and was reported in Craig et al., 2018, and was conducted in accordance with the principles of the declaration of Helsinki. The additional research ethics approval for the study reported here was granted in February 2015 (London-Hampstead Research Ethics Committee, reference 13/Lo/0482).

### Participants

Stratified sampling: thirty per cent of those who completed AVATAR therapy (15 people in total) within the AVATAR RCT [8] were invited to take part in the study. To achieve full representativeness of sample characteristics and therapy delivery, the following purposive sampling variables were applied when identifying participants: gender, ethnicity, number of voices and therapists delivering AVATAR therapy (six in total). Additionally, 5 participants who dropped out from therapy were also invited to complete the qualitative interview to explore reasons for dropping out and potential negative aspects of the therapy. All participants met inclusion and exclusion criteria for the AVATAR RCT The inclusion criteria were as follows: 1) aged over 18 years; 2) have experienced troubling auditory hallucinations for at least 12 months, and 3) primary diagnosis of non-organic psychosis (including ICD-10 categories F20-29 and F30-39, subcategories with psychotic symptoms). Criteria for exclusion were as follows: 1) CBT for psychosis or attending a group specific to hearing voices; 3) unable to identify a single dominant voice to work on; 4) refusing all medication; 5) a diagnosis of organic brain disease; 6) a primary substance dependency; 7) auditory hallucinations not in English; 8) not having sufficient English language abilities to engage in therapy and assessments, and 9) inability to tolerate the assessment process.

### Procedure

#### Interview guide

The semi-structured topic guide interview was developed in collaboration with the trial’s patient and public involvement (PPI) representative, who was also involved in the first pilot study of AVATAR therapy [6], and an independent lived experience mental health researcher. Consensus meetings with the research and therapy team facilitated the development of the interview guide.

The final interview consisted of 10 subsections including 48 questions. The subsections were as follows: reason to participate in the study (introductory question), experience of creating the “avatar”, the experience of dialoguing with the avatar, reflections on therapy sessions, use of the MP3 player, impact of the therapy on voices and everyday life, the therapist, post therapy experiences, overall experience, and software improvement.

#### Interview process

Semi-structured audio-recorded interviews were conducted by a clinical psychologist and the lived experience mental health researcher. The interviewers were not involved in therapy delivery to the present study participants. The interviews were open-ended with duration ranging from 29.11 to 58.07 min. Participants were informed that anything disclosed during the interview would not be shared with the trial therapists, and only information about significant risk would be shared with their routine care teams. Participants were paid £20 for their participation in this study. All interviews were transcribed, anonymised, and checked for accuracy.

### Analysis methods

Thematic analysis [15] was used to explore the interviews: themes and subthemes emerged after coding, categorisation and analytic reflection, in which relationships between these themes were also identified [16]. NVivo v.12 was used to support the coding, structuring, organisation, and analysis of the data. IBM SPSS 27.0 package was used to calculate the frequencies, mean and standard deviations of the demographic and clinical variables.

All transcribed data were read twice and re-read to ensure familiarity with it. Two randomly selected transcripts were preliminarily coded by two members of the research team (MRC and NE, one of whom was also involved in conducting the interviews) who independently recorded initial codes before discussing. Suggested codes were further discussed with a third team member (TW), who also reviewed the two selected interviews. As a result, a preliminary coding frame incorporating multiple perspectives on the collected data was agreed. This preliminary framework incorporated main overarching topics, mainly corresponding to the interview guide, but evolved with subsequent analysis of further interviews. When half of the interviews were coded, a reliability check was performed by an additional member of the research team (CE), which resulted in no additional codes to the previous coding frame. The resulting iteration of the coding frame was reviewed by the rest of the research team. All the research team members who contributed to the final iteration of the coding frame have a clinical psychology or psychiatry background and, with the exception of NE, they all have been trained and have delivered AVATAR therapy and have participated in data analyses of various studies of trial data (MRC, TW, CE, PAG and TC). Two independent completers of AVATAR therapy (current members of the PPI group for the new multi-centre randomised controlled trial of AVATAR therapy ISRCTN55682735, [10]) reviewed and contributed to the refinement of wording of themes and subthemes separately.

## Results

### Demographics

From the fifteen completers invited to the interview, 14 consented to participate and completed the interview (*n* = 14, 28% of completers). One person declined to take part. Their demographic and clinical characteristics are depicted in Table 1. One person who dropped out from therapy (after session 1) consented and completed the interview for the present study; the other four declined because of not being interested (3) or feeling unwell (1). Voice characteristics and reasons to take part in the AVATAR RCT are summarised in Table 2.

**Table 1:** Demographics and clinical descriptives of completers (*N* = 14)

		(N) Percentage	Mean (SD)
Age			41.93 (10.45)
Gender			
	Male	(10) 71%	
	Female	(4) 29%	
Ethnicity			
	White British	(6) 43%	
	Black British	(1)7%	
	Black Caribbean	(2)14%	
	Black African	(3)22%	
	Other	(2) 14%	
Diagnosis			
	Paranoid Schizophrenia	(11) 79%	
	Schizoaffective disorder	(2) 14%	
	Other	(1)7%	
Length of illness years			20.14 (6.7)
Number of Sessions			
	7 sessions	77%	
	8–10 sessions	21%	
	< 7 sessions^a^	2%	
Number of Voices			
	Single voice	(2)14%	
	2–5 voices	(9) 65%	
	Unsure/many	(3)21%	
PSYRATS-AH-Total (Baseline)			27.29 (4.84)

^a^ One person completed at Session 3 reporting complete cessation of voices

*Note*: *PSYRATS-AH* Positive Symptoms Rating Scale- Auditory Hallucinations

**Table 2:** Reasons for taking part in the AVATAR RCT and information about the voice selected for therapy purposes

Name (anonymised)	Age	Primary Diagnosis	Age voices started	Reason for taking part in AVATAR RCT	Sessions	Voice selected
Liam	55	Paranoid Schizophrenia	36	“I’ve been part of another study which was computerised cognitive therapy at the X Hospital.”	7	Male distressing voice
George	34	Schizoaffective Disorder	17	“I felt some kind of therapy could bring about some changes and help me to improve my life and, yeah, to seek help.”	7	Male distressing voice, “similar to myself”
Alison	35	Paranoid Schizophrenia	18	“My parents had brought it up and I thought it might be helpful and so we went from there.”	7	Female “gravelly” and abusive voice
John	52	Paranoid Schizophrenia	20	“I thought you (AVATAR team) might be able to do certain things to do to me to get rid of these demons, you know.”	10	Male “demonic” and “bullying” voice
Gregory	50	Paranoid Schizophrenia	9	“Because I thought it would benefit me to do with other things, and that the reason why is because eventually I got on, the very first time I met my therapist and I thought ‘do I really want to do this’ but I got on with her.”	7	Male “nasty” voice
William	50	Paranoid Schizophrenia	16	“Well, it just seemed a good because the NHS don’t really provide much therapy, because they can’t afford it I don’t think, so it’s just a good opportunity to get some therapy and some help with my condition really.”	7	Male “threatening” voice, “trying to attack me”
Shaheim	31	Paranoid Schizophrenia	20	“I’m hearing voices and I thought it would be helpful that the avatar can get rid of my voices.”	7	Male distressing voice
Ajay	39	Paranoid Schizophrenia	27	“Because I was hearing more than one voice, it was 4 voices, and hearing them every day is very upsetting and distressing sometimes.”	7	Male distressing voice
Assaf	33	Schizoaffective Disorder	22	“I wanted to get rid of my voices and I wanted to get better.”	7	Male distressing voice
Harry	36	Paranoid Schizophrenia	20	“My psychiatrist said to me about trying the avatar thing and I said yeah why not. And I jumped at the chance to try and do it.”	8	Male distressing voice
Imene	37	Depression with Psychotic Symptoms	34	“I actually thought it was gonna be of help to me. That it was gonna help my mental illness, because my care coordinator told me. She actually, she explained to me about it.”	7	Male “huge” and abusive voice
Mohamed	42	Paranoid Schizophrenia	25	“I decided to volunteer on the project because I used to hear voices and they disturbed me a lot.”	3 (Considered completer)	Male “big” and “abusive” voice
Anne	46	Paranoid Schizophrenia	18	“I suppose first of all, I wouldn’t have heard of it if it wasn’t referred to me by my psychiatrist. That was the first port, when he told me about it. And I thought, well it’s something different, seemed to…I liked the idea of the voices being – or the voice that we concentrated on – being acknowledged as being real, rather than just ‘oh here’s some tablets, off you go’, you know.”	7	Male distressing voice of “my deceased father”
Catherine	47	Paranoid Schizophrenia	24	“I wanted to decrease the frequency and maybe the loudness”	7	Female distressing voice, “similar to my voice, when I was a child”
Ray (Drop-out)	46	Paranoid Schizophrenia	17	“To give people like yourself insight, to be able to use, what’s the word not ulterior, different treatment strategies. If it helps other people, I’m all for these research tests.”	1	Male “dominant” and distressing voice

### Thematic analysis

**Fig. 1:**
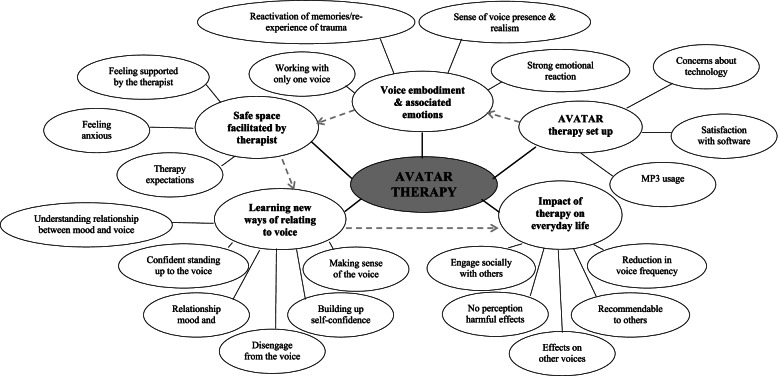
Thematic network illustrating intersection between themes

A total of 1276 references were coded. The thematic analysis identified five overarching themes: 1) AVATAR therapy set-up; 2) voice embodiment and associated emotions; 3) working in a safe space (supported by the therapist); 4) learning new ways of relating to the voices; and 5) impact of therapy on everyday life. Within these 5 themes, several sub-themes were identified by the thematic analysis (please see Fig. 1). Quotes which represent examples of each subtheme are given in Table 3.

- 1. AVATAR therapy set up
  This theme involved digital aspects of AVATAR therapy linked to engagement. Satisfaction with the software was identified as a subtheme: of those who talked about it (79%), all expressed satisfaction with the result of creating and interacting with the avatar. Concerns about technology was identified as another subtheme, with one of seven participants expressing some worries about data security or the possibility of his avatar being something found on the internet by others. The rest expressed not having concerns about using technology during sessions. Finally, all the participants shared their experience of using the MP3 with the avatar’s dialogue recordings: about half (*n* = 6) of them reported having used the MP3 1 to 4 times while being in therapy but having stopped using it at the end of it, five people reported having used it during the therapy and still “using it when necessary”, “when I feel down”, “before going to bed”, “any time that I feel like it” or “to top up” after they finished. Two people reported not having used it between sessions (only when they were with their therapist in session) but having used it after the therapy had ended. One person did not listen to the recordings at all because he was “troubled enough with his voices and did not like the sound of his own voice". Main reasons for not using it after therapy (when shared with the interviewer) were: being busy looking for a job, voice in the recording not sounding like their own voice, carrying the mp3 with them in case they need it but not having done it since end of therapy, and not needing it because “my voices hardly bother me”. All those who reported having used it either during or during and after the therapy, considered the recordings a positive resource.
- 2. Voice embodiment and associated emotions
  This theme reflects the participants views related to the process of voice embodiment and potential reactions and emotions trigged by this process. Four main subthemes emerged. Strong, and often unexpected, emotional reactions to the experience of talking back to a visual and auditory representation of the voice were shared by the majority of the participants (64%). Participants talked about feeling *anxious, frightened or embarrassed* at times. Similarly, some participants (43%) described how at times past memories emerged while conversing with the avatar; in all of these cases the memories were related to traumatic experiences (including the experience of becoming unwell with psychosis). Subjective experiences of high realism, sense of voice presence, emotional and behavioural reactions to having the avatar in front of them and interacting with it also emerged within this theme (79%). Interestingly, one participant presenting a comorbid diagnosis of Autism, expressed that for him talking to the avatar was more like talking to a computer only: “*I was satisfied, but it would have been nice if I was talking not to a computer, but to a person himself. Because I’m autistic, that’s what I was doing: talking to a computer”.* Understanding of working “with only one voice” emerged as a subtheme, with participants (43%) agreeing that it is a good approach to work initially with only one.
- 3. Safe space facilitated by therapist support
  The relationship with the therapist was highlighted by all the participants (100%), indicating that it is an essential component of AVATAR therapy. All reported feeling very supported and understood by their therapist along the sessions, and how that helped them to confront the avatar. The majority of the participants (93%) reported that talking to the avatar was challenging particularly during the first sessions: they felt anxious or uncomfortable when talking to the avatar, and even “not capable of talking back” or “forgetting what I talked to the therapist about what I would say to the avatar”. When asked about therapy expectations, one person stated that they expected that the voices “would go away permanently”, another one expressed being “sceptical” and the rest reported no specific expectations.
- 4. Learning new ways of relating to the voices
  Participants described how they acquired new strategies and confidence to talk back to their voices. Standing up to the voice, increased feelings of self-confidence when conversing with the avatar and greater sense of control over their own behaviour (i.e., choosing to engage or disengage from the voice) were three of the identified strategies. The theme also captured how participants (around 50%) explained the reduction of their distress during the course of the therapy: from high levels of anxiety at the beginning of the therapy to significantly lower levels at the end. This theme also depicted how, with the help of the therapist, AVATAR therapy helped them to make sense of their voices and how their emotions and behaviours could influence them.
- 5. Impact of therapy on everyday life
  Participants shared their experience of how their learning with AVATAR therapy affected their everyday life. Several participants (43%) highlighted how they could now engage in social situations and feel more confident around other people. The majority (80%) reported a clear reduction of the frequency of voices during their daily life and almost half of the participants talked about how the therapy impacted other voices (if multiple voices were present). All of them, except one person, reported that the other voices also became “quieter” or occurred with reduced frequency following AVATAR therapy. All completers agreed that they would recommend AVATAR therapy to other people experiencing negative distressing voice, with some of them (*n* = 3) highlighting the advantage of having a personalised image of their voice or “other” to talk to during therapy in order to gain control over the voices. Participants were also asked whether they had perceived or suffered harmful effects during or after AVATAR therapy (e.g., increase of frequency and distress of voices, agitation, low mood or hospitalisation). All those who spoke about it (86%), expressed no perception of any adverse effects or a worsening of their voices, or negative consequences in their everyday life during or after AVATAR therapy. Short term emotional reactions during therapy were captured when asked about in-session experiences (see theme *voice embodiment and associated emotions* above).

**Table 3:** Emerging themes and sub-themes of the thematic analysis

Themes	Sub-themes	Quote example	Percentage of participants
1. AVATAR therapy set-up	Concerns about technology	“And you all can find these (avatars) creeping onto the internet. It might not be anything to do with us, but somebody else will think of an avatar…”	7 (50%)
		“I was not concerned. No, not really”	
	Satisfaction with software	“It (the software) was kind of basic, but it kind of did the job, ?, I think. I don’t think it should be too complicated to be honest. I think it was just about right.”	11 (79%)
	MP3 usage	“Eventually, I started, I started listening to it more, at the beginning I didn’t feel monitored, you know.”	14 (100%)
		“I was using it around 3–4 times a week when …; at the moment I’m not using it, I don’t have my voices this much”	
		“I have enough with my voices when their troubling me, why listen to it when there not troubling me.”	
2. Voice embodiment^a^ and associated emotions	Strong emotional reaction (anxiety, fear, embarrassment)	“I was frightened of getting retaliation from my voice”	9 (64%)
		“The emotion of fear was one of the greatest, I say fear”	
	Sense of voice presence & realism	“It shook me, you know what I mean, wow. I thought wow, you know. Erm… That’s very close to the bone, do you understand what I mean?”	11 (79%)
		“Very real yeah….definitely. It used to make me jump when it first came on screen and talk to me it was like (giggles)…”	
	Reactivation of memories/re-experience of trauma	“It made me think (of) what happened to me and I was thinking maybe I might not come again because of what happened and because the session made me remember what happened to me before.”	6 (43%)
		“AVATAR (therapy) with me started off when I got ill, it went back in time, and I think that shook me with memories of the past.”	
	Working with only one voice	“I think it was sensible to only work with one… otherwise I would get confused as well.”	6 (43%)
		“I thought that was a good idea, to actually explore the voice, the main protagonist voice a little bit, and see what we could do with it.” (1310)	
3. Safe space facilitated by therapist support	Feeling supported by the therapist	“He seemed to listen, he seemed to listen quite well and he seemed to be… And he gave me a lot of positive reinforcement and told me how well I was doing, and stuff like that, which was nice. Erm, so yeah, he was quite supportive throughout the whole process, yeah”	14 (100%)
	Feeling anxious when talking to the avatar	“I used to just babble you know, just babble. Just try and say anything, because I had already forgotten what the therapist said to me, you know.”	13 (93%)
		“It was hearing the horrible words that was speaking back to me. I didn't like it. I didn't like it at all.”	
	Therapy expectations (hopes/fears for therapy)	“Exceeded them”	9 (64%)
		“No, I didn’t have any expectations at all, no. No idea, no. I just thought it was an interesting idea”	
4. Learning new ways of relating to the voices	Confident standing up to the voice	“Because the avatar started backing off, because as I started confronting it, the voice started backing off and getting less powerful, so I started getting more power over it as the sessions progressed”	10 (71%)
		“It was really great, really really helpful. Because ever since I started the avatar and even when I stopped seeing my therapist, the avatar has really helped me in the way of talking back (to my voice)”	
	Disengage from the voice	“I’m trying to get out about a bit more, because I can get a bit trapped in my own flat sometimes and everything gets too much”	4 (29%)
		“Actually, (what I) mostly did is try to ignore the voices or thinking you know, not giving back, but you know, give it a reason, you know”	
	Reduction of fear/anxiety linked to the voice	“(After the therapy) You’re not getting distressed, you’re not getting into panic, you’re not being irritated by your voices.”	7 (50%)
		“When I’m out socially and stuff, the voice, it’s a bit… it’s a lot more, erm, less stressful, like buying, shopping or going on the buses and stuff like that.”	
	Making sense of the voices	“… I suppose it’s challenged the whole idea, because one part of me says oh it’s not real, it’s just your own imagination, doing all this. But it happens all the time, and I’ve kind of begun to accept it a bit more and just get on with my life, rather than it being a problem.”	10 (71%)
		“I think afterwards I’m more watching myself. Trying to be aware of exactly what’s going on. Try to see them as less critical, yeah, and not, erm, just random voices.”	
		“I guess I am different because I now see the voices as…how do I put it? as an entertainer, that I don’t really pay that much attention to. Before I was seeing them as annoying, I was seeing them as a problem but now I don’t see them as a problem. I just see them as ‘oh you’re here again, your welcome, what can I do for you?’ just something to keep me busy.”	
	Building up self-confidence	“I’m a bit more in control, I feel a lot more confident	12 (86%)
		“It’s made me stick up for myself a bit more, erm… And a bit more generally in my everyday life, as well. Just… not just with the voices, just it’s given me a bit more confidence generally”	
	Understanding relationship between my mood and the voices	“Because it usually gets me when I’m, yeah, when I’m down or vulnerable still, but I’m kind of almost expecting it now, so I’m kind of ready for it.”	4 (29%)
		“Before, if the voice was there, my mood was low; I just wanted to be on my own or probably sleep. But now I just pick up my MP3, listen to it and that- lifts up my spirit.”	
5. Impact of therapy on everyday life	(Confidence to) Engage socially with others	“Helped me get out and reconnect with people and stuff like that”	6 (43%)
		“Socially, it has had an impact, yeah, because I feel a lot more comfortable and it *kinda* like… it feels like a lot of, being outside is a lot easier”	
	No perception of harmful events	“It’s certainly a good idea and erm more (sessions) of it won’t harm us, there’s more research information about it”	12 (86%)
		“No, because of the way it was done, like I said, because my anxiety was lessened through the sessions because, like I said, the avatar was backing off, erm, I felt safer as the sessions… I think it was quite cleverly done, the way the avatar was backing off as I was getting stronger, kind of thing. So I don’t think there’s any danger… There wasn’t, I didn’t feel like I was under danger or under threat really”	
	Reduction in frequency of the voices	“Before I had the avatar I used to hear them every day, throughout the whole day. Since I’ve done the avatar, sometimes I don’t hear them for the whole day, for a few days and then might hear them, like, throughout the day, but very, very quiet, can hardly hear.”	11 (79%)
		“There have been four or five days, or six and seven days without hearing anything at all”	
	Effects on other voices	“(It worked) With all the voices. Though mostly I hear the male one but a few times about three or four of them speaks at the same time”	6 (43%)
		“I think they’re a bit quieter, you know”	
		“It hasn’t changed the other voices at all.”	
	Recommendable to others hearing distressing voices	“It’s helped me, so probably it can help other people. And just give it a try, because it can help, it can benefit your life, I’d say.”	14 (100%)
		“Everybody’s experience is different of hearing voices. But I think having a face and voice to go with, with erm… because I think a lot of people don’t have faces to go with some of their voices, so yeah, so I think it was important to have a physical thing to interact with. So yeah”	

^a^ Embodiment- The degree to which the avatar therapy approach matched the everyday voice experience in terms of what the avatar looked/sounded and how the person felt when speaking to it

### Drop-out interview

One interview was conducted with a participant who dropped out from therapy after session 1. Ray reported hearing three voices and was experiencing psychosis for more than 29 years at the time of the interview. He also reported having had psychological therapy several times (mainly CBT) and, from his point of view, all psychological therapies that he had “helped to reduce any symptoms associated with psychosis”. His main motivation for participating was helping others, especially researchers.

During the qualitative interview, he reported not liking “being analysed” and also not liking “being watched” in general. Consequently, during his therapy session, Ray “felt an increase in paranoia, suspiciousness and anxiety”. His main reason for dropping out was the travelling to the neighbourhood where the main clinic delivering AVATAR therapy was located, to attend sessions with his therapist. He explained to the interviewer that he had previously lived in that neighbourhood and it was associated “with negative experiences” for him. The travelling to and from therapy triggered high levels of anxiety and he could only think of going back home.

Ray reported that the avatar looked “very similar to my dominant voice” and “he was very anxious about talking to it”, which he did not like. He considered that the technology was “good enough” and that he barely had to make any changes to the avatar for it to look like his voice. However, he decided that the therapy would not be good for him especially because he did not want to be in the clinic and did not want to be “analysed by the therapist or the computer”. Ray suggested that AVATAR therapy would be a good option for young people starting to suffer from psychosis.

## Discussion

The present study is, to our knowledge, the first to report the impact and personal experiences of those who have experienced AVATAR therapy. The therapy set-up, with its digital components and distinctive features in comparison with common face-to-face talking therapies, was satisfactory. The inclusion of technology was well accepted as both a means to deliver the therapy and a tool to create a digital representation of the entity behind the person’s distressing voice. The creation of the avatar and the enactment of the relationship between the person and the voice were perceived as a very helpful process to promote an engaging therapeutical dialogue. In line with Rus-Calafell et al. (2020) [11], participants reported high levels of sense of voice presence and high perceived realism of the avatar, which are proposed to contribute to the implicit activation of structures related to fear and support in vivo cognitive and emotional work. This sense of voice presence and real-time dialogue raised certain challenges identified by participants, such as emergence of strong emotional reactions and, in some cases, re-activation of trauma-related memories. However, crucially none of the participants identified any long-term negative impact related to these in-session responses consistent with the finding of no therapy-related Serious Adverse Events during the large-scale clinical trial (Craig et al. 2018) [8] . These responses are also commonly observed in trauma-focused CBT approaches [17] and potentially reflect anticipated responses to the emotional processing and rescripting of meanings associated with developmental trauma, which are central to many AVATAR therapy dialogues [5]. This finding does however underscore the importance of establishing fully informed consent with sensitive pacing and regular therapist check-ins crucial during phase 1 dialogues. The only individual factor that appeared to hinder delivery of the therapy, in terms of the avatar’s realism and on-line dialogue, was the comorbidity of autism and psychosis. The person with this comorbidity expressed clear difficulties with engaging in the dialogue with the avatar and feeling more like “talking to a computer”. Although this result must be interpreted with caution, it remains unclear whether this difficulty is best explained by the difficulties in representational and abstract thinking associated with autism, or by the inclusion of computerised social characters (the avatar) or a combination of both. Despite the fact that some studies show promising results of digital interventions for autism spectrum disorders [18], understanding the rationale of creating and dialoguing with the avatar as a representation of a social entity (‘as if’ the voice), is likely to be crucial in order for AVATAR therapy to have beneficial effects.

Participants’ views on acquisition of new strategies to deal with their distressing voices were also in line with previously identified therapeutic targets [5] and changes in relational behaviours with the avatar [14]. Improving self-confidence, (re) building sense of power and control over the voices by standing up to them, and improving their understanding (e.g., voices part of oneself, internal thoughts, association with mood) were the strategies commonly endorsed by participants. Participants’ perceived impact of AVATAR therapy on everyday life was also consistent with the RCT results [8], with most participants reporting a significant decrease of frequency of their voices. Furthermore, half of participants reported a positive impact of the therapy on how they engage with other people, suggesting generalisation of new modes of relating into daily social relationships. This converges with other relational therapies for voices (see Relating Therapy for voices [19] and Talking with Voices [2], in which the principles of assertive responding are learnt through the experience of standing up to the voices. These principles can then be generalised to difficult relationships, with the goal of voice hearers experiencing more connection to others [20].

All participants reported engaging well with the therapist and feeling supported, which helped them to confront the avatar despite, at times, feeling anxious with the experience (especially during early sessions). This positive engagement goes some way to addressing concerns about the possible negative impact on alliance of the therapist voicing the avatar (through voice transformation software). Both the consistent dialogue support and personalised positive feedback cultivated the therapeutic alliance through encouraging the person to try “new things” within a safe space. In essence the AVATAR dialogue can be understood as behavioural experiment, supported by the therapist to deliver a powerful context for new learning. Importantly a good therapeutic alliance was maintained even though the active dialogue was delivered through a digital platform whereby the participant and therapist are situated in separate rooms. This finding becomes especially relevant during covid-impacted times, suggesting the potential for AVATAR therapy to be delivered online (i.e., remotely, using video call software). This possibility would reduce the costs and practicalities of the clinic delivery while it adapts to current policies and needs of telehealth due to COVID 19 [21]. In the future, the combination of improved IT literacy and the normalisation of interacting with a computer could support greater acceptance of digital interventions, such as AVATAR therapy, and facilitate their integration into users’ daily lives.

The inclusion of the MP3 player as part of the between session activities was accepted and considered a positive resource among participants. Only one person was not able to listen to it due to the interaction with their distressing voices. Ongoing use of the MP3 following therapy completion varied across participants, with half of participants continuing to listen to recordings. The requirement to use or carry an additional device was identified as a key barrier to ongoing use, which could be mitigated in the future by access to dialogues through personal smartphones (where available) [10]. Encryption and safety related issues should be considered carefully.

One interview with a person who dropped-out from therapy was conducted. The main finding deriving from this interview was that there might had been some implications of the confluence of other psychotic experiences when delivering AVATAR therapy (e.g., persecutory delusions or difficulties with getting out of the house). Ray’s experience of “being watched and analysed during therapy” by the therapist, along with a personal history of traumatic experiences in the same neighbourhood where the mental health service was located, triggered suspicious thoughts that did not allow him to continue with the therapy. He was satisfied with the technology and managed to create his avatar, although he was not willing to have a conversation with it. These challenges appeared to be idiosyncratic as clinical characteristics of those taking part in the AVATAR RCT showed that participants presented with complex affective and non-affective psychosis (i.e., multiple voices, presence of delusions, depression and anxiety symptoms), with an average length of the illness > 20 years and common presence of trauma (see Tables 1 and 2 in [8]), and yet the majority were able to complete AVATAR therapy (71%). Further qualitative research into the experience of receiving AVATAR therapy should aim to develop a better understanding of therapy-related processes that increase the risk of drop-out.

There are some limitations of our study. Firstly, the group of therapy non-completers is clearly underrepresented. A total of 22 people dropped out from AVATAR therapy (with only 8 giving a reason for withdrawing explicitly related to therapy [5]), therefore only 5% of those who dropped out are represented in the present study (vs. the 20% of completers represented). Future qualitative evaluations of AVATAR therapy should put in place more specific procedures to keep people who dropped out of therapy engaged with the research team in the hope of learning more about their reasons for abandoning the therapy. Secondly, despite having a PPI representation in the process of creating the interview, conducting interviews, reviewing, and commenting on the coding frame, this was not a consistent group that had input into all the research procedures linked to the RCT. This limitation is being addressed in the new AVATAR multi-centre randomised controlled trial by establishing local PPI groups which contribute in a more integrated manner to the delivery of the trial in a range of ways, including the planned qualitative interview study [10]. Lastly, although there was a balance between interviewers in relation to their role in the study (a member of the research team and an external researcher who joined only to conduct interviews), socially desirable responding may have occurred to an unknown extent.

## Conclusions

This study provides important participant feedback that AVATAR therapy is acceptable and benefits participants with psychosis. All participants felt supported by their therapist, and all would recommend AVATAR therapy to others struggling with distressing voices. The thematic analysis highlighted the enablers and challenges of working dialogically with distressing voices using a digital representation, dealing with high demanding emotional, cognitive, and relational processes linked to the experience. Our analysis also identified the core strategies learnt by participants and how these were generalised to their daily life resulting into a positive change in different domains, and in particular broader social relationships.

The insights from this qualitative study together with analysis of the therapy process [5, 14] are informing the ongoing evolution of AVATAR therapy. Following the success of the first fully-powered randomised trial [8] a multi-site trial is currently underway focussing on optimisation and implementation of the approach, which includes training a cohort therapists to deliver therapy in frontline services. Future qualitative work should aim to understand factors relating to potential therapy drop-out including the opportunities and challenges of early direct work with verbatim voice content. With respect to implementation, it will also be important to explore the experiences of therapists who are being trained to deliver AVATAR therapy.

## Data Availability

Although anonymised, the qualitative datasets generated for the current study are potentially identifiable and therefore not suitable to be deposited in a public database. The data are however available from the corresponding author on reasonable request.

## Abbreviations

AVH: Auditory verbal hallucinations
AVATAR: Audio Visual Assisted Therapy Aid for Refractory auditory hallucinations
CBT: Cognitive Behavioural Therapy
IT: Information technology
NHS: National Health Service
PSYRATS-AH: Psychotic Symptoms Rating Scale Auditory Hallucinations
PPI: Patient and public involvement
RCT: Randomized controlled trial
